# Differential Expression of lncRNAs in HIV Patients with TB and HIV-TB with Anti-Retroviral Treatment

**DOI:** 10.3390/ncrna10040040

**Published:** 2024-07-13

**Authors:** Victoria A. Reid, Enrique I. Ramos, Raja Veerapandian, Areanna Carmona, Shrikanth S. Gadad, Subramanian Dhandayuthapani

**Affiliations:** 1 Center of Emphasis in Infectious Diseases, Department of Molecular and Translational Medicine, Texas Tech University Health Sciences Center El Paso, El Paso, TX 79905, USA; vreid@ttuhsc.edu (V.A.R.); r.veerapandian@ttuhsc.edu (R.V.); areanna.carmona@ttuhsc.edu (A.C.); 2 Center of Emphasis in Cancer, Department of Molecular and Translational Medicine, Texas Tech University Health Sciences Center El Paso, El Paso, TX 79905, USA; enrique.ramos@ttuhsc.edu; 3 Department of Biological Sciences, The University of Texas at El Paso, El Paso, TX 79968, USA; 4 Frederick L. Francis Graduate School of Biomedical Sciences, Texas Tech University Health Sciences Center El Paso, El Paso, TX 79905, USA

**Keywords:** transcription, gene expression, HIV-TB, TB, HIV, ART

## Abstract

Tuberculosis (TB) is the leading cause of death among people with HIV-1 infection. To improve the diagnosis and treatment of HIV-TB patients, it is important to understand the mechanisms underlying these conditions. Here, we used an integrated genomics approach to analyze and determine the lncRNAs that are dysregulated in HIV-TB patients and HIV-TB patients undergoing anti-retroviral therapy (ART) using a dataset available in the public domain. The analyses focused on the portion of the genome transcribed into non-coding transcripts, which historically have been poorly studied and received less focus. This revealed that Mtb infection in HIV prominently up-regulates the expression of long non-coding RNA (lncRNA) genes *DAAM2-AS1*, *COL4A2-AS1*, *LINC00599*, *AC008592.1*, and *CLRN1-AS1* and down-regulates the expression of lncRNAs *AC111000.4*, *AC100803.3*, *AC016168.2*, *AC245100.7*, and *LINC02073*. It also revealed that ART down-regulates the expression of some lncRNA genes (*COL4A2-AS1*, *AC079210.1*, *MFA-AS1*, and *LINC01993*) that are highly up-regulated in HIV-TB patients. Furthermore, the interrogation of the genomic regions that are associated with regulated lncRNAs showed enrichment for biological processes linked to immune pathways in TB-infected conditions. However, intriguingly, TB patients treated with ART showed completely opposite and non-overlapping pathways. Our findings suggest that lncRNAs could be used to identify critical diagnostic, prognostic, and treatment targets for HIV-TB patients.

## 1. Introduction

*Mycobacterium tuberculosis* (Mtb) is a deadly human pathogen that causes the disease tuberculosis (TB) in humans, and TB remains a severe problem in many parts of the globe. Despite the availability of effective chemotherapy, the incidence rate and the mortality rate have remained at the same level for the past several years. The World Health Organization (WHO) has recently estimated that approximately 10.5 million new cases of TB and about 1.3 million deaths due to TB occurred in the year 2022 [1]. It has also been estimated that about a quarter of the world population could harbor latent TB infection (LTBI) [2], of which about 5–10% are at risk of developing reactivation TB. The LTBI is a clinical condition where *Mtb* bacilli persist within granulomas that contain infected macrophages surrounded by non-infected macrophages, lymphocytes, and neutrophils in layers [3,4,5]. Among the various risk factors that can reactivate LTBI into active TB, such as coinfection with Human Immunodeficiency Virus 1 (HIV-1 or HIV), use of immunosuppressants like TNF-α blockers, and diabetic condition [6,7,8], HIV coinfection remains as the top risk as it has twenty times higher potential to reactivate LTBI than others [1,9,10]. 

HIV is a retrovirus that causes the disease Acquired Immunodeficiency Syndrome (AIDS) in infected people. Although it killed over two million people in the year 2004, the deaths due to HIV infection have declined recently, and it killed about six hundred and thirty thousand people in the year 2022 [11]. One of the most notorious outcomes of HIV infection in humans is the depletion of CD4^+^ T cells [12,13]. It is well established that HIV infects human immune cells through the CD4 receptor plus CCR5/CXCR4 coreceptor and that the infected cells, particularly T cells, get killed upon the multiplication and exit of the virus from the cells [14]. Paradoxically, the CD4^+^ T cells include the T helper cell type (Th1) of T cells that is critical for resisting *Mtb* infection [15,16,17]. Therefore, the infection of HIV in LTBI individuals quickly alters the immune balance that exists within the granulomas, leading to the disruption of granulomas and, eventually, the reactivation of latent bacteria to cause disseminated infection. Conversely, HIV patients are also highly susceptible to *Mtb* due to their immunodeficient conditions attributed to low CD4^+^ T cells. In addition, *Mtb* coinfection in AIDS patients exacerbates the disease by increasing the replication of HIV [13,18]. Thus, the infection of *Mtb* in HIV/AIDS patients is considered a serious risk factor, and TB appears to be the single largest cause of AIDS-related deaths (26%) [19,20]. Currently, it is estimated that at least 14 million people worldwide have HIV-*Mtb* coinfection, and these people predominantly live in countries of Africa and Asia [1]. Development of novel diagnostic and therapeutic strategies against TB in HIV patients requires an understanding of the interactions of host cells with HIV-*Mtb* coinfection. 

Several recent studies have reported that long non-coding RNAs (lncRNAs) play critical regulatory roles during host–pathogen interactions [21,22,23]. LncRNAs are non-coding RNAs with sizes of 200 nucleotides or more that have a unique ability to regulate the host cellular process by modulating the expression of protein-coding genes or their translation from mRNAs [24]. Interestingly, they also have the ability to regulate gene expression by cis-acting and trans-acting mechanisms. This feature distinguishes them from other non-coding RNAs associated with the regulation of gene expression, such as small interfering RNAs (siRNAs), microRNAs (miRNAs), and PIWI Interacting RNAs (piRNAs) [25]. Unlike small RNAs, lncRNAs can bind to a plethora of proteins in a context-dependent manner and function as decoys, guides, etc., which increases their functional diversity. The involvement of lncRNAs during host–pathogen interactions is evidenced by the altered or differential expression of lncRNA genes in host cells infected with pathogens in vitro or tissues of patients harboring infections [26,27,28,29,30,31,32,33]. It appears that the dysregulated lncRNAs modulate pathways like host immune signaling, autophagy, and apoptosis to survive and multiply within the host [22,23]. Thus, targeting the dysregulated lncRNAs remains a promising therapeutic strategy for infectious diseases. Alternatively, lncRNAs may also serve as important biomarkers for disease progression or remission.

Although the differential expression of lncRNAs has been reported in Mtb- or HIV-infected cells in vitro or the tissues of patients infected with these pathogens [34,35,36,37], lncRNA expression in HIV-TB coinfection has not been explored yet. Thus, the objective of this study was to identify differentially regulated lncRNAs in patients co-infected with HIV-TB that could play essential roles in developing treatment strategies. To accomplish this, we selected the transcriptomic dataset NCBI-GEO-GSE107104 from the public domain. This dataset included HIV and HIV-TB patients and HIV-TB patients undergoing anti-retroviral therapy (ART) from Uganda, and this dataset was previously analyzed for the expression of protein-coding genes by Verma et al. [38]. Our analysis of this dataset has revealed that the genes *DAAM2-AS1*, *COL4A2-AS1*, *AC008592.1*, *LINC00599*, and *CLRN1-AS1* were up-regulated, and *AC111000.4*, *AC100803.3*, *AC016168.2*, *AC245100.7,* and *LINC02073* were down-regulated in HIV-TB in comparison to HIV alone. Intriguingly, it also revealed that some lncRNA genes (*COL4A2-AS1*, *AC079210.1*, *MFA-AS1*, and *LINC01993*) that were up-regulated in HIV-TB patients were down-regulated in HIV-TB+ART patients, and a few lncRNA genes (*AC007922.2*, *AC111000.4, LINC01013*, and *AL139020.1*) that were down-regulated in HIV-TB patients were up-regulated in HIV-TB+ART patients. The Genomic Regions Enrichment of Annotations Tool (GREAT) analysis revealed that in a manner similar to gene expression profiles, molecular functions, biological processes, and cellular components were differentially enriched between patients with HIV-TB and HIV-TB+ART. Overall, the analyses have revealed fascinating biology and have identified specific pathways that are exclusive in nature. These findings can be leveraged to develop biomarkers and design therapeutic strategies.

## 2. Results

### 2.1. TB and ART Modulate Gene Expression Profiles in HIV Patients, including Non-Coding Transcripts

**Figure 1:**
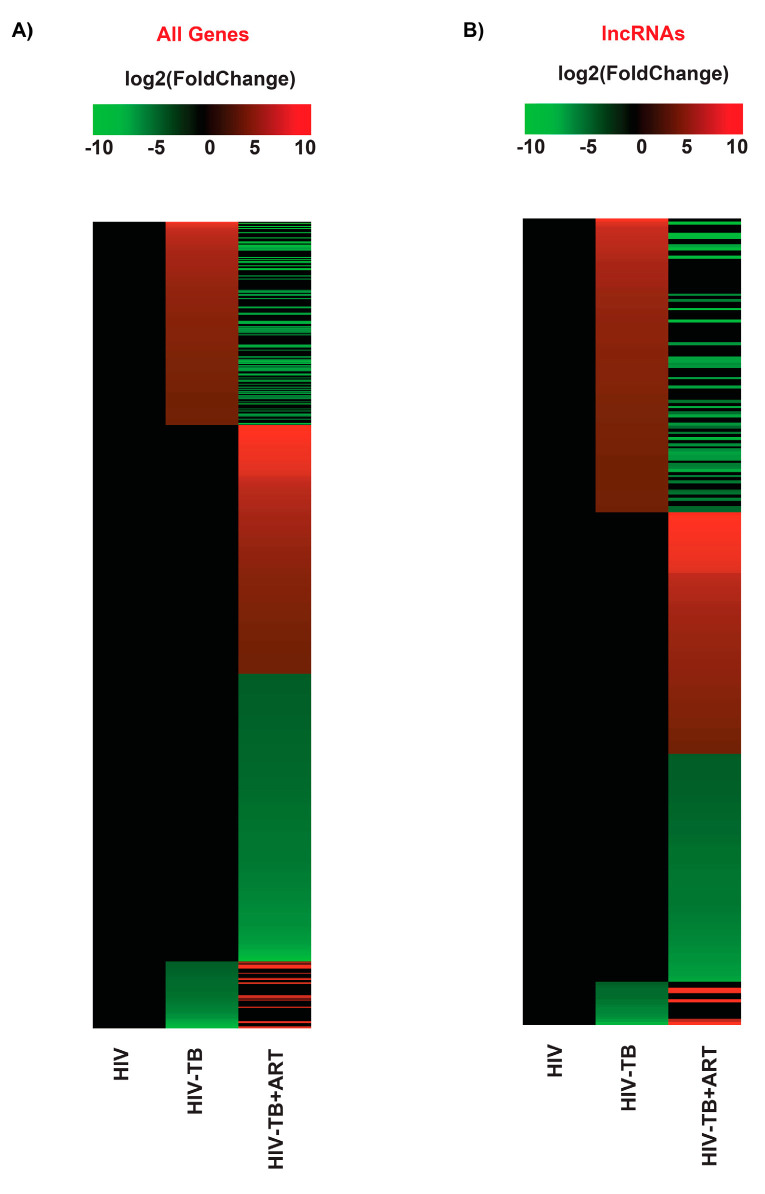
**The RNA-seq expression pattern from differentially expressed genes.** The heat map analysis on a log2(fold change) scale (–10 to 10). The gradient scale shows up-regulated (red) and down-regulated (green) regions in this study. (**A**) The heat map of all differentially expressed genes (DEGs) and (**B**) lncRNA DEGs. Samples include HIV, HIV-TB, and HIV-TB+ART.

**Figure 2:**
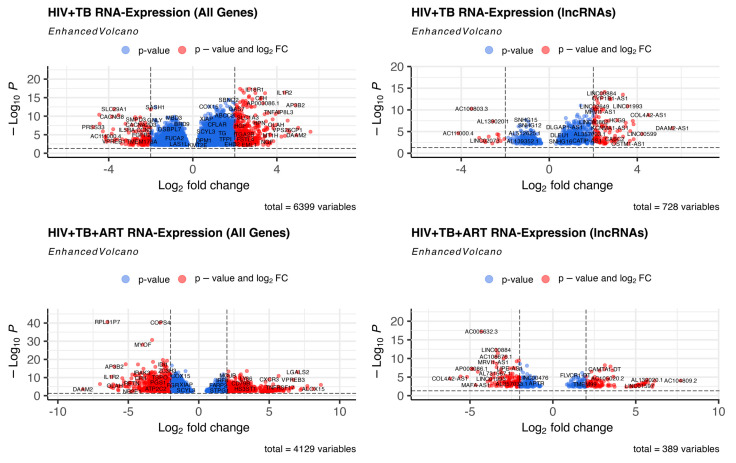
**Representation of RNA expression for HIV-TB and HIV-TB+ART.** Volcano plot for expression for all genes and lncRNAs.

**Figure 3:**
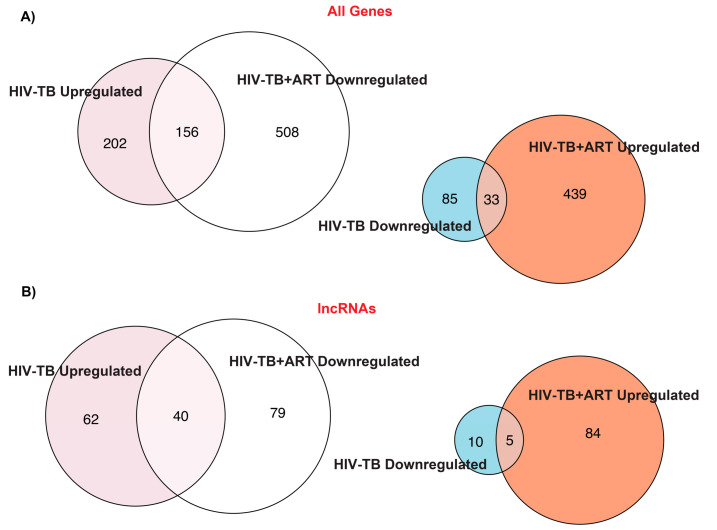
**Overlap of DEGs for HIV-TB and HIV-TB+ART.** Intersection Venn diagram analysis for (**A**) all DEGs and (**B**) lncRNA genes in HIV-TB and HIV-TB+ART samples.

Previously, Verma et al. [38] performed a whole blood RNA-seq analysis for HIV, HIV-TB, and HIV-TB+ART patients from Uganda and reported an increased expression of genes related to inflammatory pathways in HIV-TB patients. Additionally, they reported that the genes FcGR1A and BATF2 could be potential biomarkers for TB in HIV patients [38]. To identify the differentially regulated RNAs, including non-coding transcripts in this cohort, we extracted the raw RNA-seq data of this study from the GEO database, analyzed them by normalizing to HIV patients, and segregated the RNAs according to their biotypes (Figure S1). This comparison revealed a significant difference in the number of protein-coding and non-coding transcripts between the two HIV-TB and HIV-TB+ART groups compared to HIV-positive patients and differences across other biotypes, such as various pseudogenes. A heat map of all genes, including protein-coding and non-protein-coding, revealed striking differences in gene regulation among the patient groups (Figure 1, Tables S1 and S2). A volcano plot and intersectional Venn diagram of all differentially expressed genes revealed that HIV-TB patients up- and down-regulate the expression of 358 and 118 genes in relation to HIV patients, whereas HIV-TB+ART patients up- and down-regulate 472 and 664 genes in comparison to HIV patients (Figure 2 and Figure 3A, Table S1). It was also noticed that 156 of the up-regulated and 33 of the down-regulated genes from HIV-TB patients displayed opposite expression in HIV-TB+ART patients (Figure 3A, Table S1). 

Historically, the gene expression analyses focused on protein-coding genes, which are critical in HIV-TB biology. However, we examined whether, in a manner similar to mRNAs or plausibly with new roles, lncRNAs, a novel group of molecules, could be new players in this biology. A heat map exclusively presenting lncRNAs (Figure 1B) revealed that compared to the HIV patient group, 102 and 15 lncRNAs were up- and down-regulated in HIV-TB patients, while 89 and 119 were up- and down-regulated in HIV-TB+ART patients (Figure 1B and Figure 3B, Table S2). However, 40 up-regulated and 5 down-regulated lncRNAs from HIV-TB patients showed opposite regulations, respectively, in HIV-TB+ART patients. The HIV-TB+ART patients also showed some unique up- and down-regulated lncRNA genes that showed no differential expression in the HIV-TB group of patients. The top fifteen up- and down-regulated lncRNAs in HIV-TB and HIV-TB+ART in relation to HIV patients are listed in Table 1 and Table 2, respectively. A similar expression profile was also observed for protein-coding genes of these categories (Tables S3 and S4). The lncRNA genes *DAAM2-AS1*, *COL4A2-AS1*, *LINC00599*, *AC008592.1*, and *CLRN1-AS1* were the most prominently up-regulated, while *AC111000.4*, *AC100803.3*, *AC016168.2*, *AC245100.7*, and *LINC02073* were the top down-regulated lncRNAs in HIV-TB patients in comparison to HIV patients (Table 1). Similarly, lncRNA genes *AC104809.2*, *AC092068.2*, *AC007922.2*, *AC111000.4*, and *AC012511.1* were the top up-regulated, and *COL4A2-AS1*, *AC079210.1*, *AC016831.5*, *AP003086.1*, and *LINC01482* were the top down-regulated genes in HIV-TB+ART patients in comparison to HIV patients. Interestingly, the top fifteen genes up-regulated in HIV-TB+ART patients consisted of some down-regulated genes (*AC007922.2*, *AC111000.4*, *LINC01013*, and *AL139020.1*) from HIV-TB patients and the top fifteen down-regulated genes in HV-TB+ART consisted of some up-regulated genes (*COL4A2-AS1*, *AC079210.1*, *MFA-AS1*, and *LINC01993*) from HIV-TB patients. 

### 2.2. Assignment of Biological Meaning to lncRNA Genes and Predicting Their Potential Role in Various Cellular Functions

**Figure 4:**
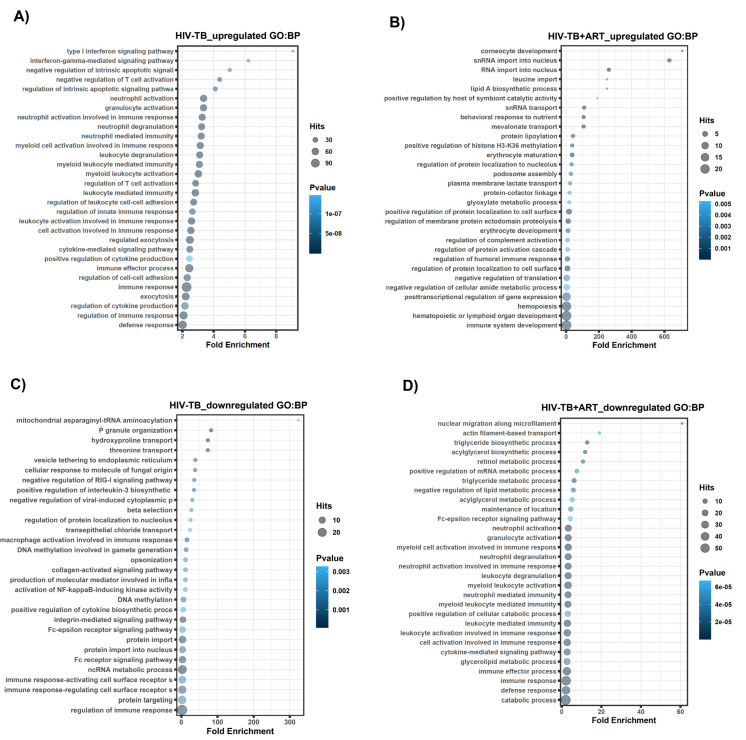
**GREAT predicts functions of cis-regulatory regions of non-coding genes for biological processes.** Analysis of non-coding regions for biological processes in HIV-TB and HIV-TB+ART. (**A**) HIV-TB up-regulated, (**B**) HIV-TB+ART up-regulated, (**C**) HIV-TB down-regulated, and (**D**) HIV-TB+ART down-regulated.

**Figure 5:**
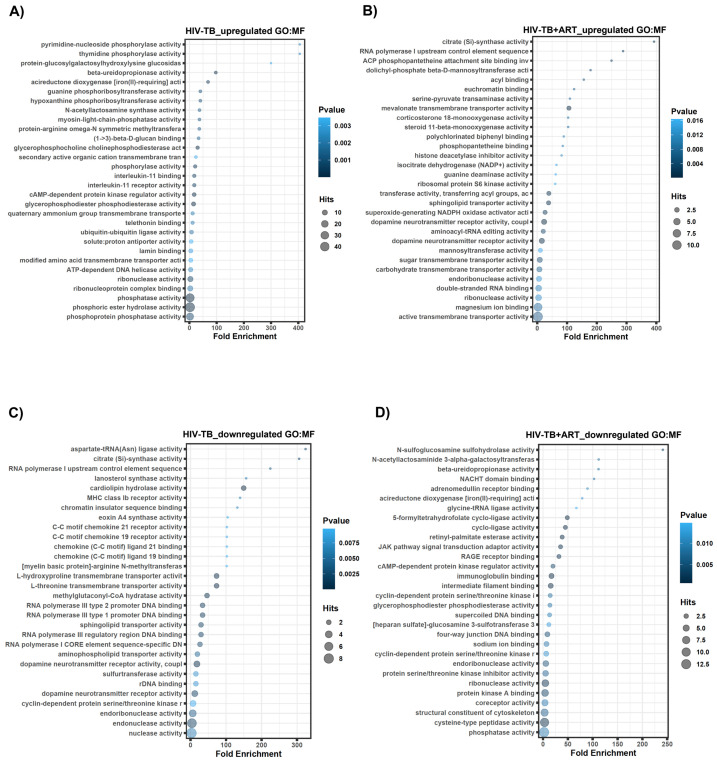
**GREAT predicts functions of cis-regulatory regions of non-coding genes for molecular functions.** Analysis of non-coding regions for molecular functions in HIV-TB and HIV-TB+ART. (**A**) HIV-TB up-regulated, (**B**) HIV-TB+ART up-regulated, (**C**) HIV-TB down-regulated, and (**D**) HIV-TB+ART down-regulated.

**Figure 6:**
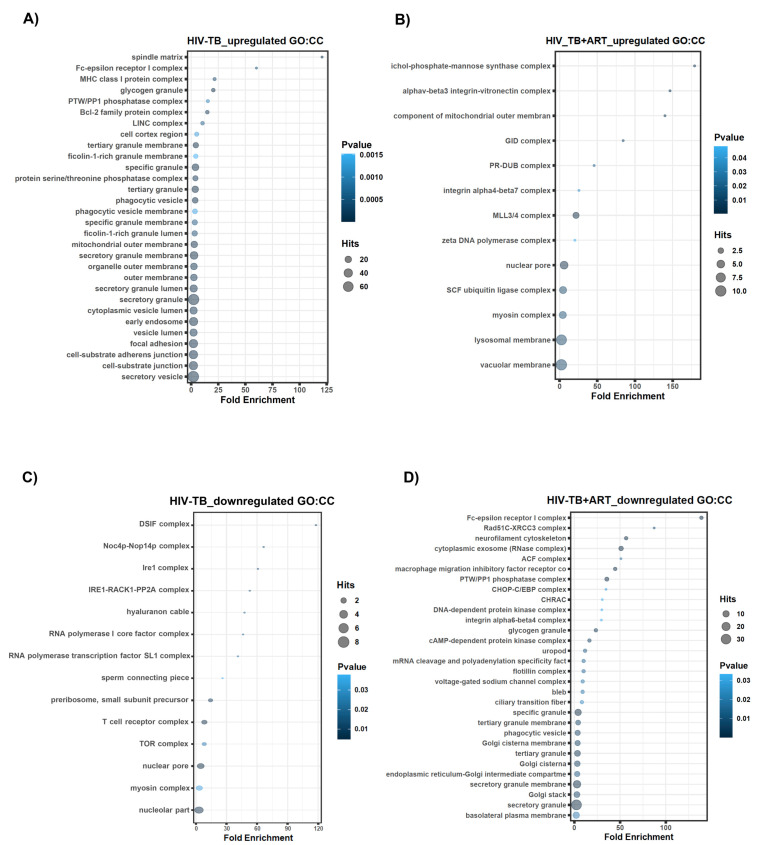
**GREAT predicts functions of cis-regulatory regions of non-coding genes for cellular components.** Analysis of non-coding regions for cellular components in HIV-TB and HIV-TB+ART. (**A**) HIV-TB up-regulated, (**B**) HIV-TB+ART up-regulated, (**C**) HIV-TB down-regulated, and (**D**) HIV-TB+ART down-regulated.

Unlike protein-coding genes, the functions of lncRNA genes are not typically well characterized. To predict the features associated with lncRNAs for each patient group, we performed a GREAT analysis of flanking regulatory regions of lncRNA loci by extracting the functional data related to nearby annotated genes. We obtained the genomic coordinates of the non-coding genes of interest via the biomaRt query. The prepared gene genomic coordinates were then uploaded to the GREAT web tool (http://great.stanford.edu, accessed on 18 October 2023) for the analysis. The input file contained the genomic coordinates of the non-coding genes, and background regions were selected as the whole genome. The GREAT analysis computes the enrichment of GO terms within the region of interest and calculates the significance of enrichment based on hypergeometric testing. The GREAT analysis showed distinct enrichment patterns for non-coding genomic regions associated with the top HIV-TB- and HIV-TB+ART-regulated lncRNA genes (Figure 4, Figure 5 and Figure 6, Tables S5–S16). Biological processes (BPs) for HIV-TB up-regulated genes were found in the main categories related to immune response regulation, exocytosis, cell–cell adhesion regulation, immune effector process, and cytokine-mediated signaling pathway (Figure 4A). On the other hand, BPs for HIV-TB+ART up-regulated genes include the following GO terms: immune system development, hematopoietic or lymphoid organ development, hemopoiesis, post-transcriptional regulation of gene expression, regulation of cellular amide metabolic process, negative regulation of translation, and regulation of protein localized to the cell surface (Figure 4B). Similarly, we analyzed the BP for HIV-TB down-regulated genes and identified the top GO terms for the regulation of the immune response, protein targeting, immune response-regulating and -activating cell surface receptors, ncRNA metabolic process, and protein import (Figure 4C). GO terms for the HIV-TB+ART down-regulated genes include the catabolic process, defense response, immune response, immune effector process, glycerolipid metabolic process, and cytokine-mediated signaling pathway (Figure 4D).

Molecular functions (MFs) were then extracted from the GREAT analysis to determine the molecular roles reasonably associated with regulated lncRNAs. Top MFs for HIV-TB up-regulated lncRNA genes include phosphoprotein phosphatase activity, phosphoric ester hydrolase activity, phosphatase activity, ribonucleoprotein complex binding, and ribonuclease activity (Figure 5A). MF GO terms associated with HIV-TB+ART up-regulated genes involve active transmembrane transporter activity, magnesium ion binding, double-stranded RNA binding, endoribonuclease activity, and carbohydrate transmembrane transporter activity (Figure 5B). Down-regulated lncRNA genes in HIV-TB samples revealed MF related to nuclease activity, endonuclease activity, cyclin-dependent protein serine/threonine kinase r, dopamine neurotransmitter receptor activity, and rDNA binding (Figure 5C). The MF GO terms associated with HIV-TB+ART down-regulated genes include phosphatase activity, cysteine-type peptidase activity, coreceptor activity, and protein kinase A binding (Figure 5D).

Additionally, we utilized the GREAT analysis to predict the possible association of genes with cellular entities, which are labeled as the cellular components (CCs) category. Our study showed that cellular structures such as the secretory vesicle, cell–substrate junction, cell–substrate adherens junction, focal adhesion, and vesicle lumen were all identified as the main categories associated with top–up-regulated lncRNA genes in HIV-TB samples (Figure 6A). Top CCs for HIV-TB+ART up-regulated lncRNA genes included regions associated with the vacuolar membrane, lysosomal membrane, myosin complex, SCF ubiquitin ligase complex, and nuclear pore (Figure 6B). Likewise, we conducted CC analyses for the HIV-TB down-regulated genes, revealing GO terms associated with the nucleolar part, myosin complex, nuclear pore, TOR complex, and T cell receptor complex (Figure 6C). Finally, the GREAT analysis of HIV-TB+ART down-regulated genes revealed an association with the following cellular entities: the basolateral plasma membrane, secretory granule, Golgi stack, secretory granule membrane, endoplasmic reticulum–Golgi intermediate compartments, and Golgi cisterna (Figure 6D).

### 2.3. Comparison of lncRNA Gene Expression Analyses among HIV-TB Cohorts between Datasets 

**Figure 7:**
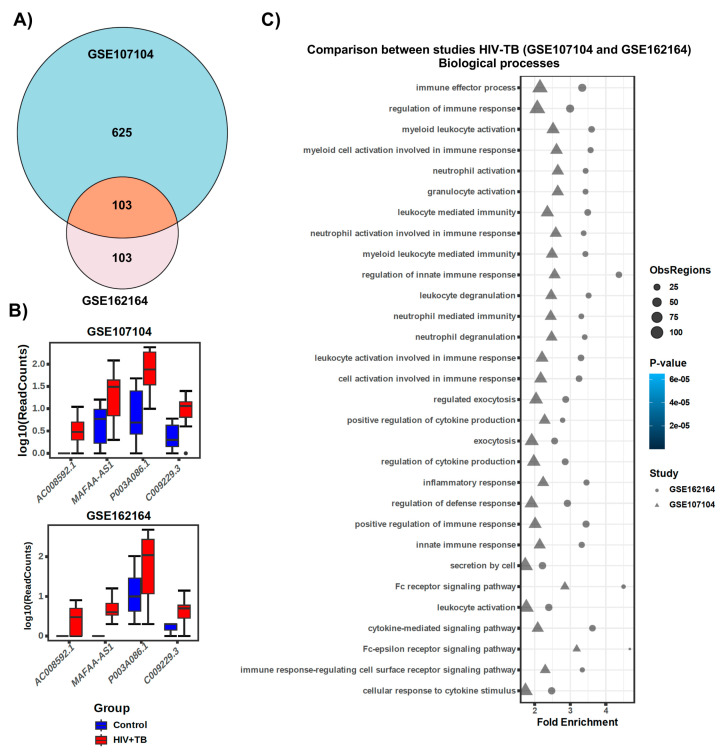
**Comparison between the two independent studies (GSE107104 and GSE162164).** (**A**) The Venn diagram showing the similarities and differences between both studies of HIV-TB. (**B**) Boxplots show the expression of candidate lncRNAs between the two studies, and the X-axis represents log10(read counts) between the Control (HIV) and HIV-TB groups. (**C**) The GREAT analysis shows a significant overlap between the different biological processes in the two studies.

To further investigate whether HIV-positive individuals with tuberculosis (HIV-TB) of this dataset (GSE107104) have similar lncRNA gene expression profiles and functions in a different study, we analyzed another (GSE162164) RNA-seq dataset [39]. Surprisingly, we found that 103 differentially regulated lncRNA genes from this dataset overlapped with the HIV-TB cohort from GSE162164 (Figure 7A). Additionally, many candidate lncRNA genes showed regulation in the same direction (Figure 7B). Furthermore, the GREAT analyses of differentially expressed lncRNA genes from the GSE162164 dataset showed significant overlap with GSE107104 for biological processes, molecular functions, and cellular components (Figure 7C and Figures S2 and S3), suggesting that the results of our analyses are reliable. However, the dataset GSE162164, as well as other cohorts in the database, did not contain RNA-seq data for an HIV-TB+ART cohort. Thus, we were unable to compare the lncRNA data for this group with that of another cohort.

## 3. Discussion

A challenging issue in HIV-TB coinfection is the atypical presentation of TB [40]. While the conventional sputum-based detection of TB is of limited scope in these patients and often leads to false negativity [41], interferon gamma (IFN-γ)-based assays, though specific [42], are more expensive. Thus, several studies have applied transcriptomics, particularly the altered or differentially expressed protein-coding genes between HIV and HIV-TB patients, to identify biological markers for TB in HIV patients [43,44]. Although initial studies have reported a large set of gene signatures, over 250 genes [44], these numbers were narrowed down to 11 (RISK11) [45] at a later time. Alternatively, an eighteen-gene signature for detecting TB in HIV patients, which was based on a meta-analysis of five microarray-based transcriptomics, was also proposed [41]. However, a recent study utilizing RNA-seq transcriptomics of two ethnic cohorts from Uganda and India has reported that genes *RAB20* and *INSL3* in peripheral blood could be used to discriminate against TB in HIV patients [39], although these markers need additional validation. 

Similar to protein-coding genes, lncRNA genes that could also be used as markers for the diagnosis and prognosis of diseases are evident from other diseases, particularly cancer, where multiple lncRNAs have been targeted for this purpose [46,47]. This prompted us to identify and analyze differentially expressed lncRNAs in HIV-TB patients. Our analysis reveals that, similar to protein-coding genes [38], several lncRNA genes are differentially expressed in HIV-TB patients with *DAAM2-AS1* and *AC016168.2* as the top up- and down-regulated lncRNAs, respectively. Interestingly, the differentially expressed lncRNAs in this study showed significant overlap with another cohort [39], suggesting that lncRNAs could be potential markers in HIV-TB. Although studies have reported the differential expression of lncRNAs from TB patients [34,35] or HIV patients [48], this is the first study analyzing all differentially expressed lncRNAs in HIV-TB patients. A previous study [49] analyzed the lncRNA expression in macrophages infected with HIV and validated four up-regulated lncRNAs in PBMCs of pulmonary TB patients. After validation, the study reported that lncRNAs *MIR3945HG V1* and *MIR3945HG V2* were up-regulated in these patients, and that they could be potential diagnostic markers in HIV-TB. However, these two lncRNAs are not among the top twenty up-regulated lncRNAs in HIV-TB samples analyzed in our study. Similarly, other studies have reported lncRNAs such as *NEAT-1*, *RIPK2*, *TGS1-1*, and *AC145676.2.1-6* from PBMCs or from whole blood samples of TB patients as potential biomarkers for TB [50]. These lncRNAs are also not seen in the list of the top twenty up- and down-regulated lncRNAs in HIV-TB, suggesting that the observed differentially expressed lncRNAs may be specific to this condition. 

A significant observation is the differentially expressed lncRNAs between HIV-TB and HIV-TB patients undergoing ART. Despite the small sample size of only two patients, the effect of ART is very discernible in this group, as it is reflected by the up- and down-regulation of a large number of lncRNA genes with significant fold differences. Similar to our results, a recent study has also noted the differential expression of lncRNAs in the PBMCs of ART-treated patients and reported that ART in HIV patients partially restores the expression of dysregulated lncRNAs [48], suggesting that lncRNAs could be possible prognostic markers in HIV and HIV-TB patients. However, a remarkable twist in the study seems to be the overlap between up- and down-regulated lncRNAs in HIV-TB and HIV-TB+ART. This encouraged us to decipher the possible roles of the lncRNAs that showed overlap between HIV-TB and HIV-TB with ART treatment. We could identify the functions, due to the infancy of the lncRNA field, for only three of them. The lncRNA gene *COL4A2-AS1,* which was down-regulated in HIV-TB+ART but up-regulated in HIV-TB, seems to be an oncogene associated with colorectal cancer [51]. It is up-regulated in colorectal cancer tissues and cell lines, and its silencing affected aerobic glycolysis but promoted apoptosis. Additionally, it was noted that *COL4A2-AS1* and hypoxia-inducible factor 1 alpha subunit (*HIF1A*) act as sponges to miR-20b-5p, indicating that it regulates gene expression through miRNA [51]. The up-regulation of this gene in HIV-TB could indicate enhanced apoptosis due to HIV infection. The second lncRNA gene with some known function is *MAFA-AS1*. This gene also showed down-regulation in HIV-TB+ART but was up-regulated in HIV-TB patients. Similar to *COL4A2-AS1*, this gene is also up-regulated in Hepatocellular Carcinoma (HCC) tissues and increases cancer progression, possibly by affecting the cell cycle by an unknown mechanism [52]. Interestingly, it is a candidate for the prognosis of HCC, perhaps suggesting its application for prognoses of patients undergoing ART. The third lncRNA gene, *LINC01013*, which is up-regulated in HIV-TB+ART but down-regulated in HIV-TB, is also associated with a cancer called anaplastic large-cell lymphoma (ALCL) [53]. It promotes the invasion of ALCL by activating the SNAIL pathway. It is plausible that *LINC01013* will activate the SNAIL pathway to produce some effect concerning the severity of the disease in this patient group. 

Further, though the functions of many of the lncRNAs in HIV-TB in this study are unknown, the GREAT analysis revealed the enrichment of genes for immune-related biological processes, notably the up-regulated lncRNA genes. Another study that analyzed the differentially expressed protein-coding genes in this cohort has also reported similar results, and these proteins are presumed to be participants of the inflammatory process by TB [38]. Consistent with this notion, this study also found elevated IFN-γ levels in the plasma of HIV-TB patients. This is not unexpected because inflammation is one of the notorious outcomes of active TB [54,55]. However, it is interesting to note that most of the enriched immune-related biological processes in the up-regulated lncRNA genes of HIV-TB were seen in the category of down-regulated genes of HIV-TB undergoing ART, raising the question of whether ART treats HIV or TB. Similarly, some up-regulated lncRNA genes that are enriched for molecular function (MF) and cellular components (CC) in the HIV-TB were down-regulated in HIV-TB patients undergoing ART. This includes lncRNA genes enriched for phosphatase activity (MF) and the secretory granule (CC). Although these observations appear to be significant, a convincing interpretation of this coincidence requires more in-depth research. 

## 4. Materials and Methods

### 4.1. Transcriptomic Data

The transcriptomic dataset for this study was accessed from NCBI-GEO-GSE107104 and -GSE162164. We have included RNA-seq data from all the patients enrolled in these studies. GSE107104 represents gene expression data from whole blood samples of 16 HIV patients, 15 HIV-TB patients, and 2 HIV-TB+ART patients from the Uganda cohort and GSE162164 represents gene expression data from 14 HIV patients and 16 HIV-TB patients from the Indian cohort. Previous publications [38,39] detailed the demographic and clinical characteristics of these patients. The demographic characteristics of the two studies are presented in Table S17. *Collection of samples from Uganda cohort:* Verma et al. [38] collected whole blood in PAXgene Blood RNA tubes and isolated RNA using the PAXgene Blood RNA Kit. They amplified the total RNA and used 100 ng of it to prepare the library. rRNAs were removed, and after mRNA fragmentation, cDNA synthesis was carried out. The double strand cDNAs were repaired and their 3’ end adenylated for adapter ligation. After ligating multiple indexing adapters, the library was enriched by PCR and validated. The qualified library was then amplified. PCR amplicons were subjected to AMPure XP bead purification and the libraries were quantified. This library was used for RNA sequencing on Illumina Hiseq2500, producing an average of 49–50 million basepair (bp) single-end reads per sample. *Collection of blood samples from Indian cohort*: Kulkarni et al. [39] collected whole blood in two PAXgene Blood RNA tubes and froze it at −80 °C. They then extracted RNA from the whole blood samples using the PAXgene Blood RNA Kit and quantified it using Qubit RNA assay HS. RNA purity was checked using QIAxpert and RNA integrity was assessed on TapeStation using RNA HS ScreenTapes. They prepared RNA libraries for sequencing using standard Illumina protocols and sequenced using the HiSeq X Ten, producing paired-end reads of 150 bp.

### 4.2. Transcriptome Assembly and Differential Expression

Sequencing of NGS paired-end reads [39] or single-end reads [38] was carried out by downloading the raw reads (fastq files) from the NCBI GEO repository using the tool SRA toolkit (prefetch and fastq-dump). Reads were inspected using the FastQC (Babraham Bioinformatics) program and trimmed for low-quality bases (Q < 20) and for adapter sequences using the program trim_galore (Babraham Bioinformatics). Alignment was performed to the human transcriptome (hg38) with annotation from Ensembl release version 101 (August 2020) using the HISAT2 aligner [56]. SAM files were converted into BAM files, followed by sorting and indexing using Samtools [57]. Read counts were computed using the alignment files and the FeatureCounts program from subread-2.0.1 package 20 [58] with reference annotation from Ensembl version 101. Read count normalization and differential gene expression analyses were calculated using the DESeq2 R package [59]. Filtering criteria for differentially expressed genes were set to a 2-fold change cutoff for non-coding genes. Visualizations from the analysis were generated in R statistical software (version 4.3.0 (2023-04-21)). The heat map was generated in R statistical software (version 4.0.3) using the package plotly.

### 4.3. Read Count Normalization from Genomic Datasets

Read count normalization using DESeq2 for RNA-seq data involved adjusting raw read counts to account for differences in sequencing depth and other technical biases across samples [59,60,61]. DESeq2 employs a method called "size factor" normalization. First, it calculates a size factor for each sample, which is the median of the ratios of observed counts to the geometric mean of counts across all samples for each gene. This size factor represents the relative sequencing depth of each sample. The raw read counts were then divided by the respective size factors, resulting in normalized counts. This normalization step ensures that the observed differences in gene expression are due to biological variation rather than technical artifacts. DESeq2 also models the count data using a negative binomial distribution, which accommodates the overdispersion typically observed in RNA-seq data, further improving the accuracy of a differential expression analysis.

### 4.4. Annotation and Composition of Non-Coding Genes

The gene list obtained from the initial analysis was subsequently queried using the Ensembl database version 101 via R statistical software (version 4.0.3) and the Bioconductor package biomaRt (interface to BioMart databases, version 2.48.0) to annotate the gene list with chromosome name, start position, end position, Ensembl gene id, external gene name, and gene biotype. Visualization plots were created using R statistical software. 

### 4.5. Genomic Regions Enrichment of Annotations Tool (GREAT) Analysis

The GREAT analysis appoints biological significance to a set of non-coding genomic regions by examining the annotations of neighboring genes. GREAT database version 4.0.4 (updated 08/19/2019) [62] was used for the analysis using the genomic coordinates (hg38) for the non-coding regions in a BED file format, using the background regions as the whole genome and standard settings from the application. The database can be accessed at http://great.stanford.edu/public/html/ (accessed on 18 October 2023). Data visualization was generated in R statistical software (version 4.0.3) using the packages ggplot2 and plotly for dot plots and heat maps, respectively. 

## 5. Conclusions

In conclusion, the differentially expressed lncRNA genes in HIV-TB and HIV-TB undergoing ART could be exploited to develop diagnostic and prognostic tools. Additional multicentric studies involving different ethnic groups will enable valuable data for such development.

## Figures and Tables

**Table 1:** List of up/down-regulated lncRNA genes in HIV-TB in relation to HIV patients.

	Up-Regulated Genes		Down-Regulated Genes	
S. No.	Gene ID	log2(Fold Change)	Gene ID	log2(Fold Change)
1	*DAAM2-AS1*	5.599065321	*AC111000.4*	−4.13637139
2	*COL4A2-AS1*	4.51193714	*AC100803.3*	−3.530569923
3	*LINC00599*	4.216277843	*AC016168.2*	−3.184558686
4	*AC008592.1*	3.835650792	*AC245100.7*	−3.054170863
5	*CLRN1-AS1*	3.831798567	*LINC02073*	−2.927654567
6	*AC016831.5*	3.797296573	*AC005277.2*	−2.903194655
7	*MAFA-AS1*	3.681848495	*AL645608.7*	−2.750602911
8	*FAM83A-AS1*	3.666535159	*LINC01996*	−2.589747063
9	*OSTM1-AS1*	3.58676319	*AL139020.1*	−2.555607503
10	*LINC01993*	3.566680578	*AC025279.1*	−2.362957614
11	*AC079210.1*	3.479032992	*AL109659.2*	−2.356363243
12	*AC021594.2*	3.461800659	*LINC01013*	−2.303911575
13	*AC010980.2*	3.353776771	*AC007922.2*	−2.299321335
14	*AP003086.1*	3.346010527	*AC008966.2*	−2.209235807
15	*AC009229.3*	3.21655715	*AC069503.1*	−2.040937496

LncRNA genes in blue color and red color were down-regulated and up-regulated in HIV-TB+ART patients, respectively.

**Table 2:** List of up/down-regulated lncRNA genes in HIV-TB+ART in relation to HIV patients.

	Up-Regulated Genes		Down-Regulated Genes	
S. No	Gene ID	log2(Fold Change)	Gene ID	log2(Fold Change)
1	*AC104809.2*	7.706535903	*COL4A2-AS1*	−6.199159707
2	*AC092068.2*	6.151251928	*AC079210.1*	−5.566015767
3	*AC007922.2*	6.043128911	*AC016831.5*	−5.175141591
4	*AC111000.4*	6.002785695	*AP003086.1*	−4.904392854
5	*AC012511.1*	5.735772764	*LINC01482*	−4.827203602
6	*AL139020.1*	5.689201588	*MAFA-AS1*	−4.647752828
7	*MIR600HG*	5.604156733	*LINC01093*	−4.528892547
8	*AL022329.2*	5.59066406	*AC005632.3*	−4.312134927
9	*AC010998.2*	5.514527786	*AL133353.1*	−3.936394158
10	*LINC01730*	5.513093748	*LINC01579*	−3.8268448
11	*CLYBL-AS1*	5.451063622	*AC103740.1*	−3.78711058
12	*AL121929.3*	5.446269531	*SCN1A-AS1*	−3.780568776
13	*LINC01013*	5.37460914	*LINC01993*	−3.726868434
14	*AC110285.2*	5.371066891	*AC093278.2*	−3.677140638
15	*LINC01597*	5.225006302	*AL078604.2*	−3.675382142

LncRNA genes in blue color and red color were down-regulated and up-regulated in HIV-TB patients, respectively.

## Data Availability

The following new datasets generated for this study are available from the NCBI’s Gene Expression Omnibus (GEO) database (http://www.ncbi.nlm.nih.gov/geo/ (accessed on 13 July 2020 and 12 June 2024) using superseries accession numbers GSE162164 and GSE107104.

## Supplementary Materials

The following supporting information can be downloaded at: https://www.mdpi.com/article/10.3390/ncrna10040040/s1, Figure S1: RNA biotype composition of RNA sequencing; Figure S2: Validation of results from GSE107104 using independent dataset GSE162164; Figure S3: Validation of results from GSE107104 using independent dataset GSE162164; Table S1: All genes; Table S2: LncRNAs; Table S3: HIV-TB protein coding; Table S4: HIV-TB+ART protein coding; Table S5: HIV-TB_up-regulated GO-BP; Table S6: HIV-TB+ART_up-regulated GO-BP; Table S7: HIV-TB_down-regulated GO-BP; Table S8: HIV-TB+ART_down-regulated GO-BP; Table S9: HIV-TB_up-regulated GO-MF; Table S10: HIV-TB+ART_up-regulated GO-MF; Table S11: HIV-TB_down-regulated GO-MF; Table S12: HIV-TB+ART_down-regulated GO-MF; Table S13: HIV-TB_up-regulated GO-CC; Table S14: HIV-TB+ART_up-regulated GO-CC; Table S15: HIV-TB_down-regulated GO-CC; Table S16: HIV-TB+ART_down-regulated GO-CC; Table S17: Demographic Characteristics.
