# Supplementary material for: Differential Expression of lncRNAs in HIV Patients with TB and HIV-TB with Anti-Retroviral Treatment
Source: Noncoding RNA. 2024 Jul 13;10(4):40. doi: 10.3390/ncrna10040040 (PMC11270221; doi:10.3390/ncrna10040040)
Supplement: Supplementary file 1 [file ncrna-10-00040-s001.zip › Reid-HIV-TB-Supplementary Materials .pdf]

Supplementary Information

"Differential expression of lncRNAs in HIV patients with TB and HIV-TB with Anti-retroviral treatment"

Reid *et al.* (2024)

This document contains the following supplementary information: Page

Supplementary Figures 2

•Figure S1. RNA Biotype Composition of RNA Sequencing…..……………………………………………………………………………..2

• Figure S2. Validation of results from GSE107104 using an independent dataset GSE162164 ………………………..…..………………………………………………………………………...3

• Figure S3. Validation of results from GSE107104 using an independent dataset GSE162164 ……………………..…………………………………………………………………………...4

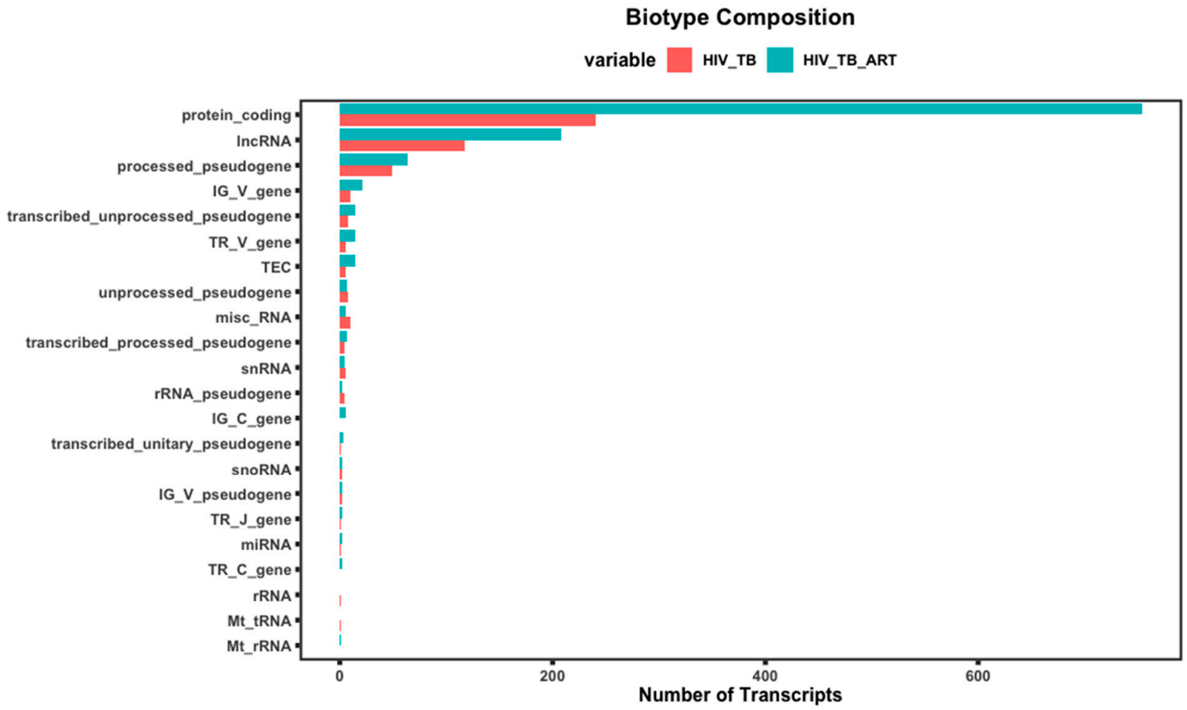

Supplemental Figure S1. RNA Biotype composition of RNA-Sequencing. This figure depicts the distribution of various RNA biotypes, including mRNA, miRNA, lncRNA, and others, obtained through RNA-Sequencing of samples from patients with HIV-TB and HIV-TB +ART. Each segment of the chart represents the proportional abundance of a specific RNA biotype, illustrating differences in expression or composition between the conditions. Notably, the data predominantly consist of sequences from protein-coding genes, lncRNA genes, and processed pseudogenes.

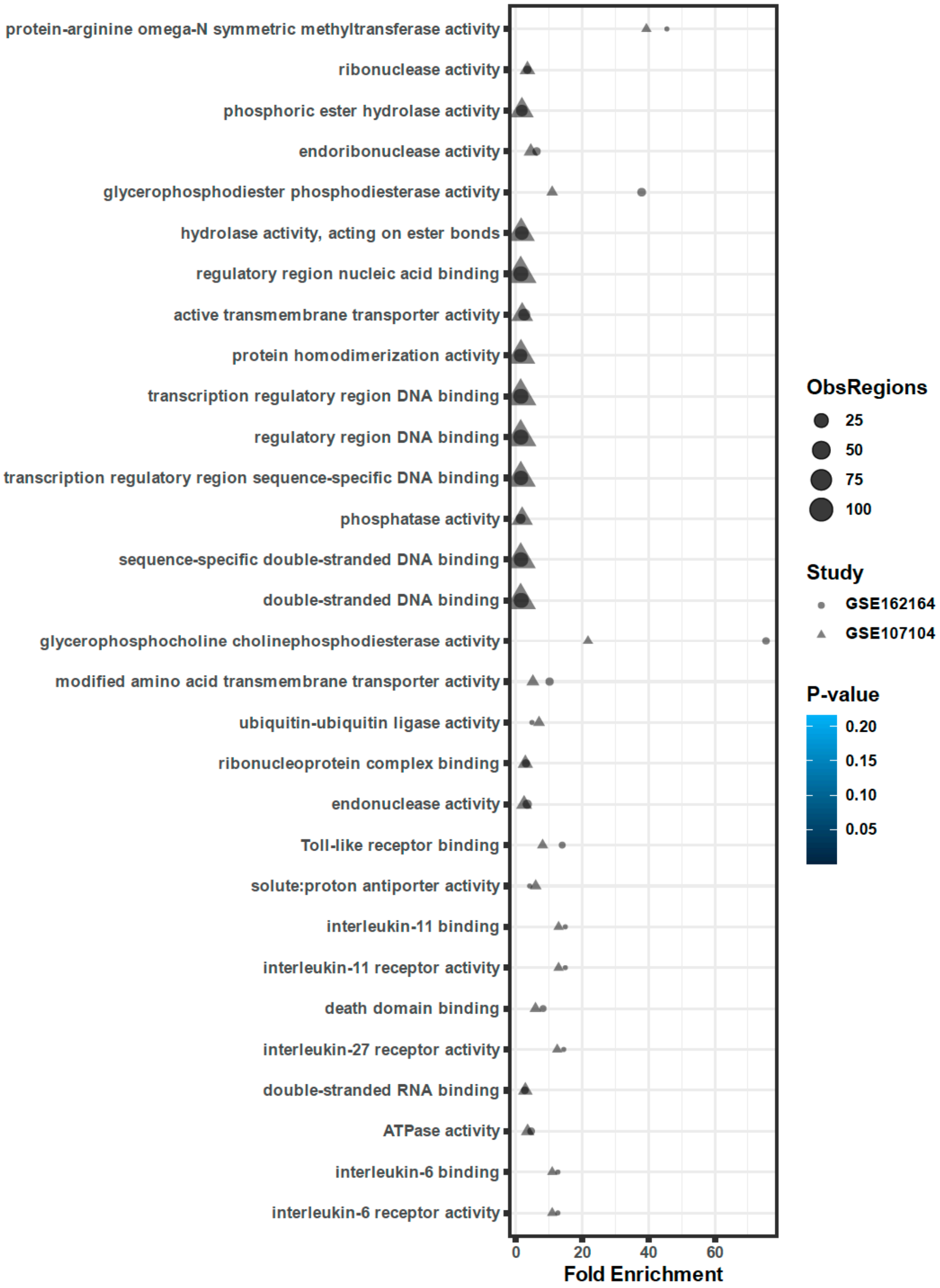

Supplemental Figure S2. Validation of results from GSE107104 using an independent dataset GSE162164. GREAT analysis shows a significant overlap between the different molecular functions in the two studies.

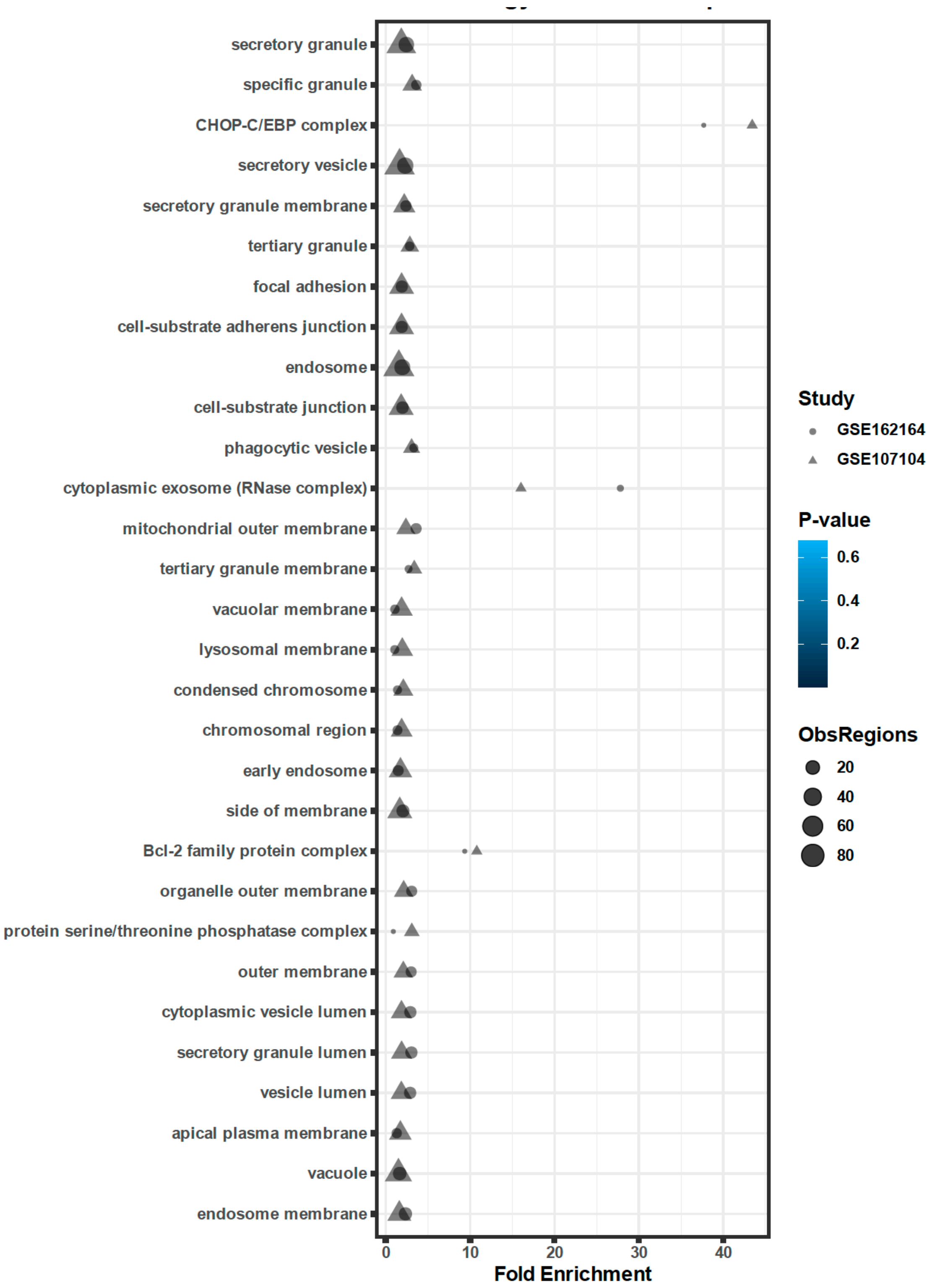

Supplemental Figure S3. Validation of results from GSE107104 using an independent dataset GSE162164. GREAT analysis shows a significant overlap between the different cellular components in the two studies.
