# Supplementary material for: Differential Expression of lncRNAs in HIV Patients with TB and HIV-TB with Anti-Retroviral Treatment
Source: Noncoding RNA. 2024 Jul 13;10(4):40. doi: 10.3390/ncrna10040040 (PMC11270221; doi:10.3390/ncrna10040040)
Supplement: Supplementary file 1 [file ncrna-10-00040-s001.zip › Table S3.pdf]

Table S3. List of upregulated/down-regulated protein-coding genes in HIV-TB in relation to HIV patients.

	Up-regulated genes		Down-regulated genes	
**S. No**	**Gene ID**	**Log_2 Fold change**	**Gene ID**	**Log_2 Fold change**
**1**	*DAAM2*	4.929216367	PRSS33	-4.733780503
**2**	*AP3B2*	4.88489983	CCL23	-4.450881309
**3**	*IL1R2*	4.367018771	SIGLEC8	-4.431394049
**4**	*TNFAIP8L3*	4.084537592	CACNG6	-4.278305311
**5**	*OLAH*	3.961524129	ALOX15	-3.856659631
**6**	*MT1H*	3.689263679	CACNG8	-3.844867449
**7**	*ZSCAN4*	3.688787162	PTGDR2	-3.786489276
**8**	*CHMP4C*	3.547649174	VPREB1	-3.751076508
**9**	*ZDHHC19*	3.544046052	SLC29A1	-3.740939755
**10**	*NGF*	3.533645501	PRSS41	-3.59468982
**11**	*CLEC2A*	3.503292858	FOXO6	-3.362895434
**12**	*ITGA7*	3.480441621	PLAAT5	-3.202633984
**13**	*CYP1B1*	3.437595227	PCDH8	-3.198966551
**14**	*EPB41L4B*	3.43059924	IL5RA	-3.134451126
**15**	*FAM20A*	3.415367253	RNASE3	-2.884879942
**16**	*EPPK1*	3.38436271	GNG3	-2.880783075
**17**	*METTL7B*	3.378321259	ARC	-2.869946399
**18**	*CFH*	3.246794276	MYBPC2	-2.834580628
**19**	*VNN1*	3.230247966	HTR3A	-2.826822781
**20**	*RD3L*	3.19270553	SYCP2L	-2.813571122
