# Supplementary material for: Differential Expression of lncRNAs in HIV Patients with TB and HIV-TB with Anti-Retroviral Treatment
Source: Noncoding RNA. 2024 Jul 13;10(4):40. doi: 10.3390/ncrna10040040 (PMC11270221; doi:10.3390/ncrna10040040)
Supplement: Supplementary file 1 [file ncrna-10-00040-s001.zip › Table S4.pdf]

Table S4. List of upregulated/down-regulated protein-coding genes in HIV-TB+ART patients in relation to HIV patients.

	Up-regulated genes		Down-regulated genes	
**S. No**	**Gene ID**	**Log_2 Fold change**	**Gene ID**	**Log_2 Fold change**
**1**	*ALOX15*	8.129851654	*DAAM2*	-8.222773546
**2**	*CACNG6*	7.215184207	*CD177*	-6.298356528
**3**	*LGALS2*	7.035459271	*IL1R2*	-6.185016978
**4**	*VPREB3*	6.706244573	*CYP19A1*	-6.073428276
**5**	*PLD4*	6.630918411	*ZDHHC19*	-6.067393918
**6**	*SYCP2L*	5.909146645	*AP3B2*	-5.992579128
**7**	*BEND5*	5.791043985	*OLAH*	-5.965246592
**8**	*TNFRSF17*	5.776072064	*EPPK1*	-5.73645616
**9**	*ZG16B*	5.734111002	*OR2C3*	-5.701617801
**10**	*USP41*	5.71527528	*PCSK9*	-5.698897535
**11**	*TCL1A*	5.666438373	*TNFAIP8L3*	-5.426470941
**12**	*CD8B2*	5.658925049	*MCEMP1*	-5.319807971
**13**	*ZNF683*	5.64071501	*FAM20A*	-5.305185854
**14**	*DNASE1L3*	5.636936906	*ANKRD34B*	-5.260052566
**15**	*TRNP1*	5.289617916	*S100A12*	-5.184451877
**16**	*NIPAL2*	5.284659711	*MMP8*	-5.057452808
**17**	*RTL5*	5.262766234	*PCOLCE2*	-4.957671923
**18**	*FAM53A*	5.251542893	*GPR84*	-4.902190457
**19**	*OVGP1*	5.156996691	*RD3L*	-4.851068146
**20**	*TM4SF19*	5.124663434	*ITGA7*	-4.779767242

Protein-coding genes in blue and red color were down-regulated and upregulated in HIV-TB patients, respectively.
